# PEGylation increases antitumoral activity of arginine deiminase of *Streptococcus pyogenes*

**DOI:** 10.1007/s00253-021-11728-7

**Published:** 2021-12-15

**Authors:** Rico Schwarz, Eric Zitzow, Adina Fiebig, Silvio Hering, Yvonne Humboldt, Nina Schoenwaelder, Neele Kämpfer, Kerren Volkmar, Burkhard Hinz, Bernd Kreikemeyer, Claudia Maletzki, Tomas Fiedler

**Affiliations:** 1 Institute of Pharmacology and Toxicology, Rostock University Medical Centre, Schillingallee 70, 18057 Rostock, Germany; 2 Institute of Medical Microbiology, Virology, and Hygiene, Rostock University Medical Centre, Schillingallee 70, 18057 Rostock, Germany; 3 Department of Medicine, Clinic III - Hematology, Oncology, Palliative Medicine, Rostock University Medical Center, Ernst-Heydemann-Str. 6, 18057 Rostock, Germany; 4 Present Address: Division of Immunology, Paul-Ehrlich-Institute, Langen, Germany

**Keywords:** Arginine deiminase, PEGylation, Glioblastoma multiforme, Cancer therapy

## Abstract

**Abstract:**

Arginine auxotrophy is a metabolic defect that renders tumor cells vulnerable towards arginine-depleting substances, such as arginine deiminase (ADI) from *Streptococcus pyogenes* (SpyADI). Previously, we confirmed SpyADI susceptibility on patient-derived *glioblastoma multiforme* (GBM) models in vitro and in vivo. For application in patients, serum half-life of the enzyme has to be increased and immunogenicity needs to be reduced. For this purpose, we conjugated the *S. pyogenes-*derived SpyADI with 20 kDa polyethylene glycol (PEG20) moieties, achieving a PEGylation of seven to eight of the 26 accessible primary amines of the SpyADI. The PEGylation reduced the overall activity of the enzyme by about 50% without affecting the Michaelis constant for arginine. PEGylation did not increase serum stability of SpyADI in vitro, but led to a longer-lasting reduction of plasma arginine levels in mice. Furthermore, SpyADI-PEG20 showed a higher antitumoral capacity towards GBM cells in vitro than the native enzyme.

**Key points:**

• *PEGylation has no effect on the affinity of SpyADI for arginine*

• *PEGylation increases the antitumoral effects of SpyADI on GBM in vitro*

• *PEGylation prolongs plasma arginine depletion by SpyADI in mice*

## Introduction

Arginine is a semi-essential amino acid required for the biosynthesis of proteins, polyamines, creatinine, and nitric oxides. Furthermore, the availability of arginine is important for a variety of biological pathways, e.g., cell growth, proliferation, and survival (Delage et al. 2012; Husson et al. 2003; Morris 2004, 2009; Riess et al. 2018; Wu et al. 2009; Wu and Morris 1998). Human cells obtain arginine either from extracellular or intracellular sources. Extracellular arginine originates from diet or is provided by the kidney where the majority of the circulating arginine is synthesized. Extracellular arginine is taken up into the cell via solute carrier proteins (SLC) (Fultang et al. 2016). Intracellularly, arginine is obtained by proteolytic degradation and arginine de novo synthesis in the urea cycle. It has been observed that cancer cells frequently downregulate non-essential biosynthesis pathways to allow faster growth with smaller energy expenditure (DeBerardinis et al. 2008). Accordingly, in several tumor entities including hepatocellular carcinoma, prostate or pancreatic cancer, GBM, and others, arginine auxotrophy has been found (Bowles et al. 2008; Fiedler et al. 2015; Kim et al. 2009; Syed et al. 2013; Wu et al. 2013). The arginine auxotrophy is caused by a defective arginine synthesis due to the inactivation of the arginine succinate synthetase 1 gene (*ASS1*) or the arginine succinate lyase gene (*ASL*) by hypermethylation of CpG islands in the promoter regions (Delage et al. 2012; Fiedler et al. 2015; Fultang et al. 2016; Riess et al. 2018; Syed et al. 2013). Arginine auxotrophy makes these cancer cells prone for arginine depletion-based therapies.

The susceptibility of cancer cells to arginine deprivation was first observed in cancer cell cultures contaminated with *Mycoplasma spp*., as the bacteria efficiently utilize arginine for energy production via the arginine deiminase system (ADS) (Kenny and Pollock 1963; Kraemer 1964; Kraemer et al. 1963; Schimke et al. 1966). The ADS comprises the enzymes arginine deiminase (ADI), ornithine carbamoyltransferase, and carbamate kinase and is present in numerous bacteria. The ADS is an important catabolic pathway in bacteria and has been shown to contribute to their energy metabolism, as ATP is produced upon the ADS mediated degradation of arginine (Cunin et al. 1986; Deibel 1964; Fiedler et al. 2011; Hering et al. 2013). Furthermore, the ADS contributes to intracellular pH homeostasis in acidic environments, e.g., in several streptococci (Casiano-Colon and Marquis 1988; Cusumano and Caparon 2015), by providing (a) ammonia to capture protons and (b) additional ATP that drives F0F1 type ATPases to pump protons out of the cytosol (Levering et al. 2016; Ryan et al. 2009). The primary step in the ADS pathway is the ADI mediated deimination of L-arginine to L-citrulline and ammonium.

The use of ADI as a therapeutic drug was first proposed by Miyazaki et al. (1990) and the efficacy was demonstrated for multiple tumor entities (for review, see Riess et al. 2018). Currently, recombinant ADI from *Mycoplasma spp.* is the most frequently used enzyme for arginine deprivation therapy (Ensor et al. 2002; Holtsberg et al. 2002; Tomlinson et al. 2015). Drawbacks of (*Mycoplasma*-derived) ADI are the high immunogenicity and the rapid renal clearance due to the low molecular weight of 35 kDa. Both problems can partially be circumvented by PEGylation (Delage et al. 2010; Fultang et al. 2016). PEGylation is the conjugation of biomolecules with polyethylene glycol (PEG). PEGylated enzymes have a vastly decreased immunogenicity and enhanced serum half-life while retaining most of their enzymatic activity. This was first described by Abuchowski and colleagues in 1977 for bovine liver catalase (Abuchowski et al. 1977a). PEG is commonly linked to proteins via a coupling agent in a two-step reaction. In a first step, PEG reacts with the coupling agent forming activated PEG. In a second step, the activated PEG reacts with a functional group, e.g., an amino group or sulfhydryl group. During this reaction, the coupling agent is either substituted by the protein (e.g., *N*-succinimidyl ester) or reacts directly with the protein, thereby acting as a linker between protein and PEG (Abuchowski et al. 1977b). Activated PEG reagents of different functionalities and molecular weights, utilizing a great variety of different linking chemistries, are commercially available today (Abuchowski et al. 1977b; Bailon and Won 2009; Jevsevar et al. 2010).

To date, all arginine-depleting therapies in clinical trials are based on an ADI derived from *Mycoplasma spp*. For clinical application, the enzyme is usually conjugated PEG moieties with a molecular weight of 20 kDa (ADI-PEG20) (Holtsberg et al. 2002). In this form, it can be applied to ASS1 negative and thus arginine auxotrophic tumors. ASS1 loss has even become a reliable predictive and prognostic biomarker for poor outcomes (Szlosarek et al. 2021). Hence, ADI-PEG20 should be used further in clinical settings.

We previously proposed ADI from *Streptococcus pyogenes* (SpyADI) as an alternative to *Mycoplasma spp*.-derived ADI, as it is well adapted to the conditions of the human body. Furthermore, its higher molecular weight of 46 kDa suggests better pharmacokinetic properties than *Mycoplasma-*derived ADI (Fiedler et al. 2015; Hering et al. 2013). We could previously show that heterologously produced and purified SpyADI efficiently inhibits the growth of hepatocellular carcinoma and GBM tumor cells in vitro and in an ectopic xenograft mouse model (Fiedler et al. 2015; Maletzki et al. 2017).

Here, we describe the PEGylation and purification of the heterologously expressed SpyADI and its properties and antitumoral activity in comparison to the native enzyme.

## Material and methods

### Bacterial strains and culture conditions

For heterologous expression of the ADI of *S. pyogenes* serotype M49 strain 591 (SpyADI), the previously described *Escherichia coli* DH5α clone carrying the plasmid pASK_IBA2_ArcA was used (Hering et al. 2013). The *E. coli* strain was cultivated at 37 °C under ambient air conditions in Lysogeny Broth medium containing 100 mg/l ampicillin (LB_Amp_).

### GBM cell lines and treatment schedule

Previously described low-passage glioblastoma cell lines HROG02, HROG05, HROG52, and HROG63 were used in this study (Fiedler et al. 2015; Maletzki et al. 2017). Cells were cultured in DMEM/F12 medium supplemented with 10% fetal calf serum (FCS) and 6 mM L-glutamine at 37 °C in a 5% CO_2_ enriched humidified atmosphere. For SpyADI treatment, 5 × 10^3^ cells/well were seeded in triplicates in a 96-well plate and incubated in standard medium for 37 °C. Subsequently, cells were treated for up to 72 h with 35 mU/ml SpyADI or SpyADI-PEG20 in standard medium.

### Calcein AM staining

For biomass quantification of GBM cells in 96-well plates, cells were stained with Calcein AM. For that purpose, cells were washed with 100 µl PBS per well and then incubated with 100 µl PBS containing 2 µM Calcein AM. After incubation for 20 min at 37 °C, fluorescence was measured with a SpectraMax M3 microplate reader with excitation at 485 nm and emission at 535 nm. Biomasses of treated cells were related to biomasses of untreated control cells.

### Heterologous production of SpyADI

*S. pyogenes* ADI was heterologously produced in *E. coli* as described before (Fiedler et al. 2015; Hering et al. 2013; Maletzki et al. 2017). For SpyADI production, 50 ml of LB_Amp_ medium were inoculated with *E. coli* DH5α pASK_IBA2_ArcA cells from an LB_Amp_-agar plate and incubated overnight at 37 °C under vigorous shaking. The next day, two Erlenmeyer flasks each containing 500 ml of LB_Amp_ medium were pre-warmed to approximately 37 °C and inoculated with 25 ml of the pre-culture. The bacteria were incubated at 37 °C under vigorous shaking until an optical density at 600 nm of 0.5–0.6 was reached. The SpyADI production was induced by the addition of anhydrotetracycline (final concentration 0.2 µg/ml). The bacteria were further incubated overnight at room temperature on a laboratory shaker. Subsequently, both cultures were pooled and the cells were harvested by centrifugation at 4000 g for 20 min and stored at − 20 °C.

### StrepTactin affinity chromatography

Protein purification was achieved by affinity chromatography on StrepTactin sepharose as described previously (Feldman-Salit et al. 2013; Hering et al. 2013). In brief, bacterial pellets were thawed, suspended in 5 ml buffer W (100 mM Tris–HCl pH 8.0, 1 mM EDTA, 150 mM NaCl) and cell disruption was achieved by the FastPrep method with acid-washed glass beads. Two cycles of 30 s each at a speed of 6.0 ms^−1^ were applied. In between, samples were cooled on ice for at least 2 min. Cell debris was removed by centrifugation for 10 min at 20,000 g. Afterwards, the clear supernatant was loaded on a column containing 1 ml StrepTactin sepharose (IBA Lifesciences) equilibrated with 2 ml of buffer W. The column was washed five times with 1 ml of buffer W and the SpyADI was eluted with 6 to 8 times 500 µl buffer W containing 2.5 mM desthiobiotin. Each elution fraction was collected in a separate 1.5 ml micro-reaction tube for SDS-PAGE and Western blot analysis. Subsequently, all fractions containing the desired protein were pooled and dialyzed for 16 h in 800 ml PBS buffer at pH 6.8 or pH 8.0 (for PEGylation) using a dialysis tube with a molecular weight cut-off (MWCO) of 15 kDa (Serva Electrophoresis GmbH). The dialyzed sample was then either used for PEGylation or subjected to an Amicon ultra centrifugal filter tube (Merck KGaA) with a MWCO of 30 kDa and centrifuged for 10 min at 2300 g and 4 °C to increase protein concentration of the sample. Subsequently, the activity was determined and the SpyADI solution was stored at 4 °C.

### PEGylation

The protein concentration of a SpyADI preparation after dialysis in PBS at pH 8.0 was determined by Bradford analysis and adjusted to a concentration of 1 mg/ml with PBS (pH 8.0). Then a 40-fold molar excess of PEG reagent (mPEG20K-Succinimidyl Carboxymethyl Ester, Sigma Aldrich) was dissolved in 1–2 ml PBS (pH 8.0). For example, for PEGylation of 1 mg SpyADI (MW: 49,783 g/mol), 16 mg PEGylation reagent (MW 20,000 g/mol) were added. PEG reagent and SpyADI were mixed and incubated for 2 h at room temperature with stirring. Afterwards, the reaction mixture was dialyzed against 1 × PBS (pH 6.9) overnight at 4 °C. Subsequently, PEGylated SpyADI was separated from native SpyADI by anion exchange chromatography.

### Anion exchange chromatography

Separation of PEGylated from native SpyADI was achieved by anion exchange chromatography using a strong anion exchange column HiTrap Q XL 1 ml (GE Healthcare). First, all buffers were degassed by filtration through a 0.22 µm filter. The column was washed with five column volumes of start buffer/mobile phase A (12 mM phosphate buffer, pH 7.4, containing 2.7 mM KCl and 10 mM NaCl) using a syringe that was connected to the column via a Luer lock connector. Subsequently, the column was washed with five column volumes of regeneration buffer/mobile phase B (12 mM phosphate buffer, pH 7.4, containing 2.7 mM KCl and 1 M NaCl) and equilibrated with ten column volumes of start buffer. The sample was loaded onto the column and the flow through was collected. The column was then washed with five column volumes of start buffer and again the flow through was collected. The elution was done with a LC-20AD HPLC system (Shimadzu) at a flow rate of 0.2 ml/min for 3 h applying a linear NaCl concentration gradient from 10 to 500 mM in 12 mM phosphate buffer (pH 7.4, containing, 2.7 mM KCl) and elution fractions were collected in 1-ml portions. After initial assessment of the appropriate NaCl concentration for the elution of SpyADI-PEG20, the elution was done in a 2-step gradient including 45 min elution with 210 mM NaCl (elution of SpyADI-PEG20) followed by 15 min elution with 500 mM (elution of native SpyADI) at a flow rate of 0.2 ml/min.

### Arginine deiminase activity assay

ADI activity was determined as described before (Hering et al. 2013). In short, ADI activity was measured by quantifying the ammonia released during the conversion of L-arginine to L-citrulline. The standard assay mixture had a final volume of 250 µl and contained 100 mM MES/KOH (pH 6.5), 5 mM MgCl_2_, and 10 mM L-arginine and 50 µl of ADI sample (undiluted or diluted 1:10, 1:20, 1:50, 1:100, 1:200, 1:400, and 1:800 in distilled water). The reaction was started by the addition of L-arginine. For kinetic measurements, L-arginine was added in the range from 0.5 to 20 mM. After 10–30 min at 37 °C, the amount of ammonia in the assay mixture was determined with the Rapid Ammonia Assay Kit (Megazyme). The activity was calculated as U/ml or U/mg protein and 1 Unit was defined as conversion of 1 µmol L-arginine per minute.

### Arginine quantification

For quantification of arginine concentration, an Agilent 1260 Infinity II HPLC system with a Diode Array Detector (DAD) G7117A was used. The system was controlled by OpenLAB CDS Workstation software. For sample preparation, 100 µl 35% perchloric acid were added to 1 ml sample, mixed, and placed on melting ice for 10 min. After the precipitation step, 55 µl potassium hydroxide solution (7 M) were added and centrifuged for 2 min at 20,000 g. The supernatant was filtered through a 0.22 µm syringe filter into a HPLC sample vial.

Separation of arginine from other substances in the sample was performed by using a reversed-phase column (Agilent Poroshell 120 EC-C18 4.6 × 100 mm, 2.7 µm). The standard L-arginine (Sigma-Aldrich, St. Louis, MO, USA) was dissolved in deionized water and used in concentrations of 1 µM–1 mM. The quantification of arginine was done according to manufacturer’s guidelines (AdvanceBio Amino Acid Analysis, © Agilent Technologies, Inc. 2018).

### Determination of accessible primary amines

Free primary amines were determined by 2,4,6-trinitrobenzene sulfonic acid (TNSBA) staining. For that purpose, 120 µl of protein solutions with concentrations between 20 and 200 µg/ml were incubated with 60 µl TNSBA (0.01% [w/V]) in 0.1 M NaHCO_3_ (pH 8.5) in 96-well plates for 2 h at 37 °C. The reaction was stopped by adding 60 µl SDS (10% w/V) and 30 µl HCl (1 N) per sample. Subsequently, absorbance was measured at 335 nm in a SpectraMax M3 (Molecular Devices) microplate reader. The number of accessible primary amines in proteins was calculated based on a standard curve with L-alanine at concentrations of 2–20 µg/ml.

### Serum stability

Venous blood from four healthy donors was drawn into 7.5 ml serum Monovettes (Sarstedt) and cell-free supernatants were obtained after sedimentation. Serum from all four donors was pooled, and stored at − 80 °C. Written informed consent was obtained according to the local Ethics Committee (reference number A2017-0191) and the guidelines for the use of human material. To assess serum stability of SpyADI and SpyADI-PEG20, 200 µl enzyme solution with 2 mg/ml were mixed with 600 µl human serum and incubated at 37 °C for up to 48 h. As controls, (1.) 200 µl of SpyADI or SpyADI-PEG20 were mixed with 600 µl PBS and (2.) 600 µl serum were mixed with 200 µl PBS and treated equally. At different time points, 30-µl samples were taken, immediately mixed with SDS sample buffer, and stored on ice. Samples were subjected to SDS-PAGE and Western blot analysis with alkaline phosphatase conjugated StrepTactin to detect SpyADI and carboxyterminal degradation fragments. Furthermore, samples were taken for ADI activity measurements.

### Ethical statement

All animal experiments were approved by the local governmental authority (approval number: 7221.3–1-032/19–3), in accordance with the governmental animal protection law and the EU Guideline 2010/63/EU. Therefore, 6-week-old female NOD.Cg-Prkdc^scid^Il2rg^tm1Wjl^ (NSG, Charles River Laboratories, Lyon, France) mice were used as recipients. Mice were bred in the local animal core facility under specific pathogen‐free conditions. During the experiment, mice were kept in type III cages (Zoonlab GmbH, Castrop‐Rauxel, Germany) at 12‐h dark:light cycle, the temperature of 21 ± 2 °C, and relative humidity of 60 ± 20% with food (pellets, 10 mm, ssniff‐Spezialdiäten GmbH, Soest, Germany) and tap water ad libitum. All animals received enrichment as mouse-igloos (ANT Tierhaltungsbedarf, Buxtehude, Germany), nesting material (shredded tissue paper, Verbandmittel GmbH, Frankenberg, Deutschland), paper roles (75 × 38 mm, H 0528–151, ssniff‐Spezialdiäten GmbH), and wooden sticks (40 × 16 × 10 mm, Abedd, Vienna, Austria).

### Pharmacodynamics of SpyADI and SpyADI-PEG20 in vivo

Twenty-seven NSG mice were randomly divided into three groups with nine animals each. One group was intravenously injected in the tail vein with 250 U/kg body weight of SpyADI, the second group was injected with the same activity of SpyADI-PEG20, and the third group was injected with PBS as a control. Blood was collected 6 h, 12 h, or 24 h after injection by retro-orbital bleeding. EDTA was added as anti-coagulant. Plasma was obtained by centrifugation for 10 min at 13,000 g. Plasma arginine levels were determined via HPLC as described above.

## Results

**Fig. 1:**
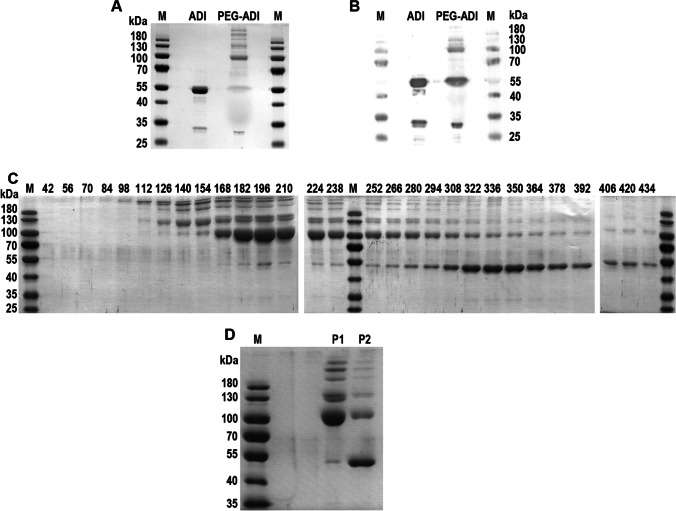
SpyADI PEGylation and purification. **A** Coomassie-stained SDS-PAGE gel and **B** Western blot showing M = marker, ADI = purified unpegylated SpyADI, ADI-PEG = SpyADI after the PEGylation reaction; **C** Coomassie-stained SDS-PAGE gel of elution fractions of the anion exchange chromatography for the separation of SpyADI and SpyADI-PEG20, numbers above the lanes = NaCl concentration the elution started with in the respective fraction (each fraction covers a 14 mM NaCl concentration range), M = marker; **D** Coomassie-stained SDS-PAGE gel showing M = marker, P1 = pooled and concentrated fractions eluted with 160 to 240 mM NaCl, P2 = pooled and concentrated fractions eluted with 295 to 390 mM NaCl

**Fig. 2:**
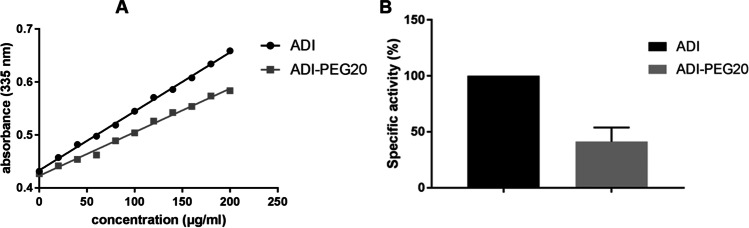
PEGylation efficiency and impact on activity. **A** Concentration dependent absorbance of TNBSA stained SpyADI and SpyADI-PEG20 (means of *n* = 5 biological replicates). As described by Holtsberg et al. (2002), the molar PEGylation rate was calculated as *x* = (1 − (slope PEG-ADI / slope ADI)) × 26, where 26 refers to the number of accessible primary amino groups of SpyADI. **B** Relative specific activity of SpyADI-PEG20 in comparison to native SpyADI, means with standard deviations of *n* = 4 biological replicates

Heterologous production and purification of SpyADI yielded 7.1 ± 1.5 mg pure enzyme per liter of culture (*n* = 4 biological replicates). The purified SpyADI was PEGylated with a 40-fold molar excess of mPEG20K-Succinimidyl Carboxymethyl ester for 2 h at room temperature and dialyzed in PBS pH 7 for neutralization. The PEGylation success was analyzed via SDS-PAGE and Western blot (Fig. 1A and B). The majority of the SpyADI was conjugated with PEG20 residues, mostly resulting in a protein with an apparent molecular weight of 100 kDa, but variants with 120 till > 180 kDa occurred as well. Some of the SpyADI however remained unmodified (55 kDa signal). In order to separate PEGylated SpyADI from the remaining unmodified SpyADI, anion exchange chromatography was applied. Initially, a NaCl gradient from 10 to 500 mM was used for the elution to assess the appropriate NaCl concentration to recover PEGylated ADI from the column. The majority of the PEGylated SpyADI eluted at NaCl concentrations from 160 to 240 mM (Fig. 1C, lanes 10–15). The native SpyADI was mainly released from the column at NaCl concentrations between 295 and 390 mM (Fig. 1C, lanes 20–26). Therefore, for the preparation of SpyADI-PEG20 for subsequent experiments, elution from the anion exchange column was carried out with a NaCl concentration of 210 mM. The fractions containing the PEGylated SpyADI were pooled and concentrated by ultrafiltration, yielding an almost pure SpyADI-PEG20 preparation (Fig. 1D). This yielded 3.6 ± 1.0 mg SpyADI-PEG20 from 1 l of initial expression culture (*n* = 4 biological replicates). PEGylation efficiency was determined by TNBSA staining of the accessible primary amines in the native and the PEGylated SpyADI. As determined using a standard curve generated with L-alanine, the native SpyADI contains 26 accessible primary amino groups. To determine the degree of PEGylation of the SpyADI-PEG20, different concentrations of SpyADI and SpyADI-PEG20 were stained with TNBSA (Fig. 2A) and the molar ratio of PEG/SpyADI was calculated as described in Holtsberg et al. (2002). With our PEGylation approach, in average, 7.5 (± 1.7, *n* = 5) mole of PEG were conjugated with one mole of SpyADI. Measurement of the enzymatic activity of the SpyADI-PEG20 in comparison to the native SpyADI revealed that this degree of PEGylation reduces the specific activity of the enzyme to about 50% as compared to the unmodified enzyme (Fig. 2B).

**Fig. 3:**
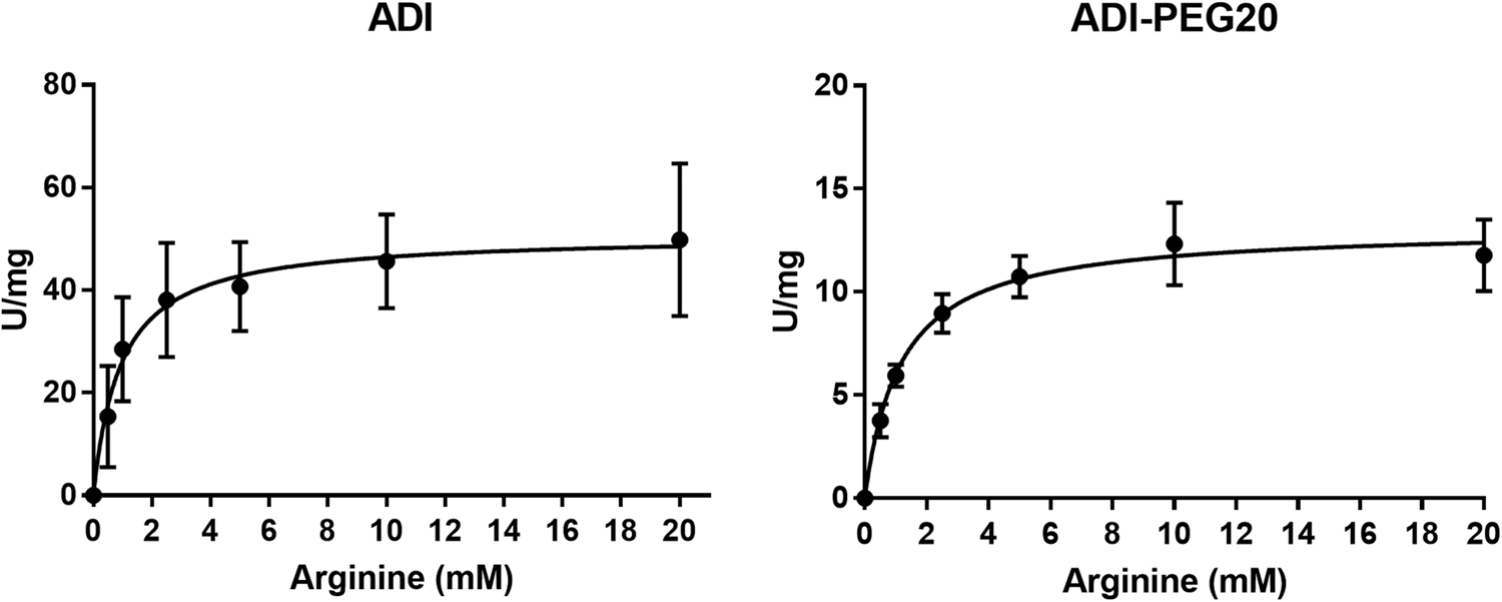
Michaelis–Menten kinetics. Activity of SpyADI and SpyADI-PEG20 is shown as a function of arginine concentration. Means with standard deviations of *n* = 3 biological replicates

Although overall specific activity of SpyADI was reduced by PEGylation, the binding affinity of the enzyme to arginine was not significantly affected, as Michaelis–Menten kinetics with the specific activity of the enzyme as a function of the arginine concentration revealed Michaelis constants (Km) of 0.94 ± 0.3 and 1.04 ± 0.3 mM arginine for native and PEGylated SpyADI, respectively (Fig. 3).

**Fig. 4:**
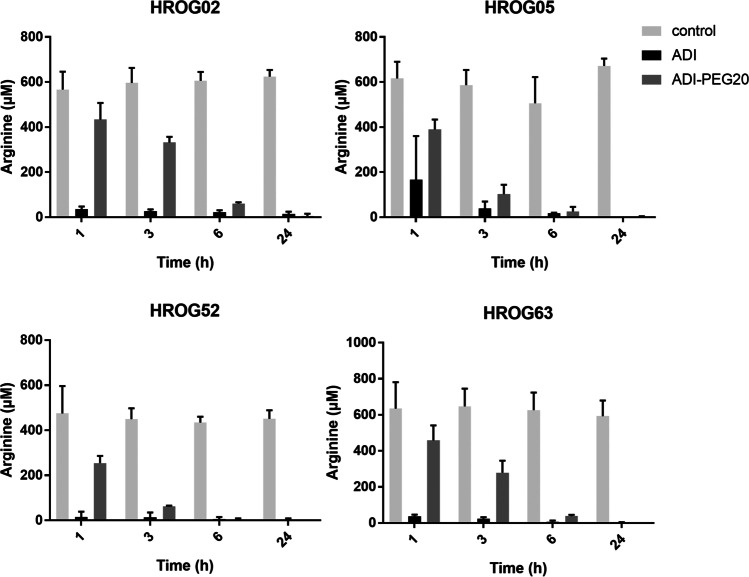
Arginine concentrations in culture supernatants. GBM cells were treated with 35 mU/ml of either SpyADI or SpyADI-PEG20 in standard medium for 24 h. Untreated cells served as a control. Arginine concentrations in the culture supernatant were measured after 1, 3, 6, and 24 h. Bars and whiskers represent means and standard deviation of *n* = 3 biological replicates

Next, we analyzed the efficiency of arginine depletion of native SpyADI and SpyADI-PEG20 in vitro. For that purpose, the GBM cell lines HROG02, HROG05, HROG52, and HROG63 were treated with 35 mU/ml SpyADI or SpyADI-PEG20 in standard medium. Untreated cells of all cell lines served as a control. The arginine concentration in the supernatant of the cells was determined 1 h, 3 h, 6 h, and 24 h after application of the SpyADI variants. Both SpyADI and SpyADI-PEG20 reduced the arginine concentrations in the medium to below 10 µM in all cell lines (Fig. 4). Under these conditions, the arginine concentrations decreased more quickly in supernatants of cells treated with the native SpyADI. After 24 h, however, arginine concentrations were lower in supernatants of the SpyADI-PEG20-treated HROG02, HROG52, and HROG63 cells than in the SpyADI-treated cells. In the latter two cases, arginine levels dropped below the detection limit 24 h after application of the SpyADI-PEG20 (Fig. 4).

**Fig. 5:**
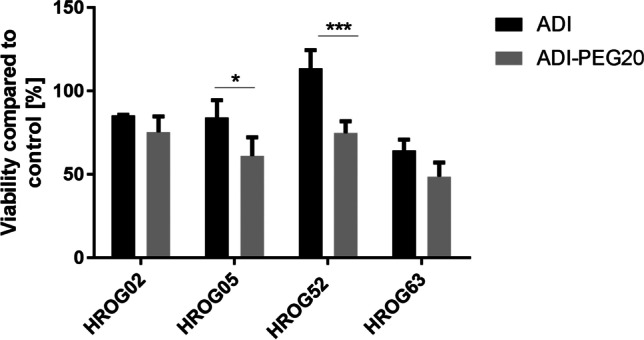
Antitumoral activity of SpyADI or SpyADI-PEG20. GBM cells were treated with 35 mU SpyADI or SpyADI-PEG20 for 72 h. Biomass was assessed by Calcein AM staining and absorbance measurement at 570 nm. Absorbance measured for untreated control cells was set to 100% and the other values were related, respectively. Shown are means and standard deviations of *n* ≥ 3 biological replicates, *p < 0.05, ***p < 0.001, two-way ANOVA

We previously showed that *S. pyogenes* SpyADI efficiently inhibits growth of GBM cells in vitro and in an ectopic xenograft mouse model (Fiedler et al. 2015; Maletzki et al. 2017). To assess the impact of PEGylation on the antitumoral effect of SpyADI, 35 mU/ml of native SpyADI or SpyADI-PEG20 were applied to four different low-passage human GBM cell lines for 72 h in vitro. Native SpyADI reduced the biomass of the GBM lines HROG02, HROG05, and HROG63 to 60–80% as compared to the untreated control (Fig. 5). For HROG02 and HROG63, the biomass reduction caused by the SpyADI-PEG20 was slightly and for HROG05 significantly stronger than the reduction caused by the unmodified SpyADI. The GBM line HROG52, however, was not responsive to treatment with native SpyADI at all, but showed a significant reduction in biomass to 75% of the untreated control when treated with SpyADI-PEG20.

**Fig. 6:**
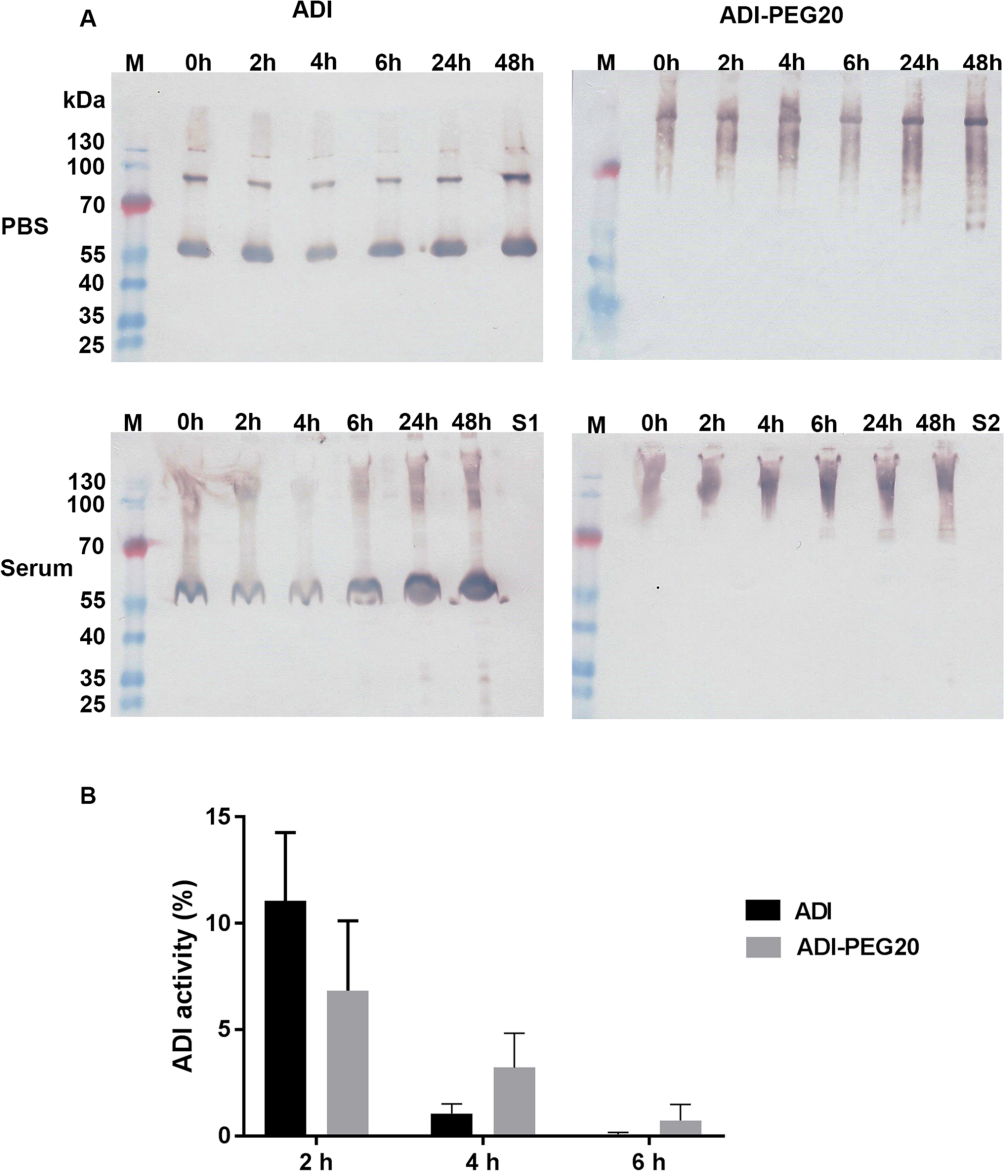
Stability in human serum. **A** Western blot analysis of SpyADI (left column) and SpyADI-PEG20 (right column) after incubation for up to 48 h in PBS (upper row) and human serum (lower row). M = marker (Page-RulerTM Plus Prestained Protein Ladder), S1 = fresh pure serum, S2 = pure serum after 48 h of incubation at 37 °C. **B** Enzymatic activity of SpyADI and SpyADI-PEG20 after 2, 4, and 6 h incubation in human serum at 37 °C in % of the specific activity at the start of the incubation. Means and standard deviations of *n* = 3 biological replicates, no significant differences, two-way ANOVA

To assess whether the enzyme is stable in human serum, native SpyADI and SpyADI-PEG20 were incubated with serum pooled from four healthy donors for up to 48 h. Proteolytic degradation was analyzed via SDS-PAGE, Western blot (Fig. 6A), and enzymatic activity (Fig. 6B). While no major proteolytic degradation was detectable for both native SpyADI and SpyADI-PEG20 for at least 24 h, the activity of both forms of the enzyme dropped quickly after exposure to the serum, resulting in loss of about 90% of the activity within the first 2 h for both native SpyADI and SpyADI-PEG20. After 4 h and 6 h of incubation, the remaining activity of the SpyADI-PEG20 was slightly higher than the activity of the native enzyme but below 5% of the initial activity for both (Fig. 6B).

**Fig. 7:**
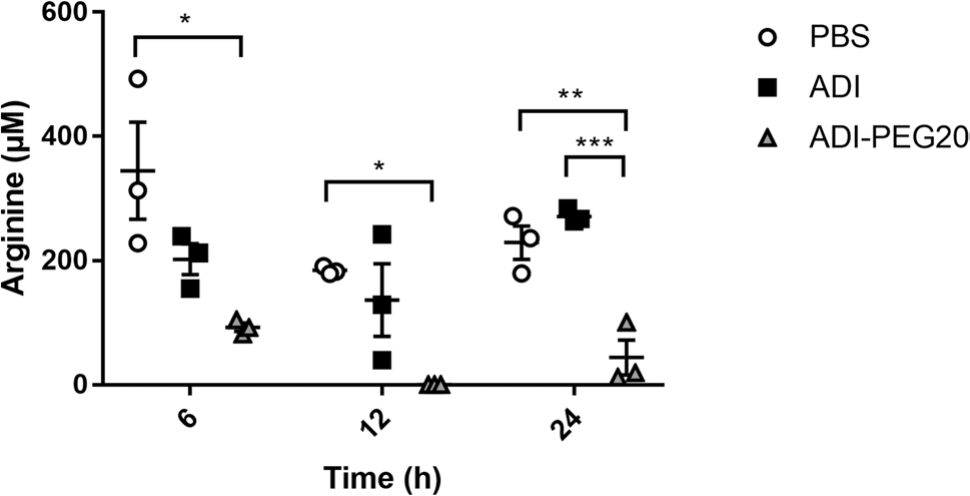
Plasma arginine concentrations in mice. Mice were injected with 250 U/kg body weight of SpyADI or SpyADI-PEG20. PBS served as a mock control. Plasma arginine concentrations were determined 6, 12, and 24 h after injection. Each symbol represents one individual. Horizontal lines and vertical whiskers represent means and standard error of means. *p ≤ 0.05, **p ≤ 0.005, ***p ≤ 0.001, one-way ANOVA

When injected into mice, the arginine level in the murine plasma was more efficiently reduced by the SpyADI-PEG20 than by the unmodified SpyADI at each time point measured (Fig. 7). Twelve hours after intravenous application, the plasma arginine was reduced below the detection limit in the SpyADI-PEG20-treated mice. Twenty-four hours post injection, the arginine concentration in the plasma of mice treated with unmodified SpyADI was at the same level as in the PBS-treated control animals. In SpyADI-PEG20-treated mice, however, the arginine level was still significantly reduced in comparison to mice receiving PBS or unmodified SpyADI (Fig. 7).

## Discussion

The improved pharmacokinetic properties of PEGylated proteins are due to the masking of the immunogenic peptides with the non-immunogenic PEG without causing conformational changes in PEGylated proteins (Digilio et al. 2003; Hinds and Kim 2002; Wang et al. 2002; Youngster et al. 2002). The reason for the low immunogenicity of PEG itself is unknown, but is generally attributed to its simple structure (Schellekens et al. 2013).

While the reduction of the immunogenicity is mostly independent from the molecular weight of the conjugated PEG, the serum half-life of PEGylated proteins increases with the molecular weight of the PEG. This is due to the inhibited renal clearance by glomerular filtration in the kidney. The inhibition increases with the increasing Stokes radius of PEG molecules starting at a threshold of 70 Å (20 kDa PEG). This threshold effectively represents the maximum size of PEG in the modification of biopharmaceuticals, as there is an inverse relationship between specific activity and PEG size (Bailon and Won 2009).

Since the streptococcal ADI does not contain any primary amino group bearing amino acids in the active site, an amino group-specific PEGylation reagent was applied. The approach used here leads to random PEGylation of primary unprotonated (nucleophilic) amino groups. Secondary amino groups are not accessible. It has been described, however, that PEGylation of primary amino groups is not random, but there is some selectivity towards ε-amino groups of lysine residues (Bailon and Won 2009; Jevsevar et al. 2010; Roberts et al. 2002). The number of accessible amino groups determined for the SpyADI resembles that described for the Mycoplasma ADI (Holtsberg et al. 2002). We could show that in average, about 30% of the TNBSA stainable amino groups of the SpyADI were PEGylated with our approach.

After PEGylation, the SpyADI retained about 50% of its initial specific activity, which is in the range reported for other PEGylated enzymes, e.g., *Mycoplasma*-derived ADI with roughly the same PEGylation rate (Holtsberg et al. 2002).

When the same activity (35 mU/ml) of SpyADI-PEG20 and native SpyADI is applied to GBM cells in vitro, the antitumoral effect of SpyADI-PEG20 was significantly higher in HROG05 and HROG52. In the other two GBM lines, a tendency towards a stronger effect was detected as well. In our experiments, we monitored arginine levels in culture supernatants over a period of 24 h. In a direct comparison of the two SpyADI preparations, the native form depleted arginine faster than the SpyADI-PEG 20. In the case of HROG02, HROG52, and HROG63, the stronger antitumoral effect might be explained by the lower arginine concentrations in the medium of the SpyADI-PEG20-treated cells after 24 h in comparison to those treated with the native SpyADI. In HROG05, however, the arginine concentration was below the detection limit in the medium of cells treated with the native SpyADI, while residual arginine (1.7 µM) was detected in the supernatant of SpyADI-PEG20-treated cells. Why the effect of the SpyADI-PEG20 on viability of the cells was still more pronounced is unclear. It can be speculated that a subpopulation of the HROG05 cells regains the ability to produce arginine upon the arginine depletion pressure. The SpyADI-PEG20 might be active for a longer time as compared to the native SpyADI and be able to degrade the de novo synthesized arginine in the 72 h incubation period. This may have boosted toxicity characterized by autophagy, senescence, and necrosis (Fiedler et al. 2015; Maletzki et al. 2017). All four cell lines tested have previously been shown to be arginine auxotrophic due to epigenetic silencing of genes of the urea cycle (Fiedler et al. 2015; Maletzki et al. 2017). However, HROG52 cells did not respond to the native SpyADI, consistent with previous studies describing the same unexplained phenomenon for these cells (Maletzki et al. 2017). Although the exact reason for the enhanced antitumoral activity of the SpyADI-PEG20 in vitro remains elusive, we assume the better stability compared to its native form as underlying cause. This would be in line with the plasma arginine levels measured in mice after application of native SpyADI and SpyADI-PEG20 to the animals. Here, plasma arginine levels increased again after an initial drop following the application of the native SpyADI, while arginine levels in the plasma of mice injected with SpyADI-PEG20 remained low 24 h post application. Similar effects were reported by Holtsberg et al. (2002) for mice injected with native and PEGylated ADI from *Mycoplasma sp*., were plasma arginine levels remained close to the detection limit for 6 days post injection of an ADI-PEG20 variant, while 24 h post injection of native Mycoplasma ADI more than 60 µM of arginine was detected in the plasma of the animals (Holtsberg et al. 2002).

Our findings argue in favor of using the PEGylated SpyADI not only for future in vivo but also for in vitro approaches.

Finally, our optimized PEGylation process described here constitutes an ideal starting point for combination approaches with cytostatic or even immunomodulatory agents. Indeed, several clinical trials are currently assessing the impact of ADI-PEG 20 to chemotherapy or immune-checkpoint inhibition (Harding et al. 2021) (*ClinicalTrials.gov identifier NCT02709512*). A very recent phase 1 study holds promise for the latter because of increased intratumoral T cell infiltration induced by ADI-PEG 20 (Chang et al. 2021). This is of particular importance for tumors with a low immunogenicity, such as GBM. Prospective follow-up studies will show whether this combined approach provides a real treatment option for cancer patients.

## Data Availability

The datasets generated during and/or analyzed during the current study are available from the corresponding author on reasonable request.
